# Impact of a virtual reality-enhanced learning program for maternal positioning in midwifery students: an exploratory multicenter pre–post study

**DOI:** 10.3389/fmed.2026.1771624

**Published:** 2026-03-06

**Authors:** Carla Sa-Couto, Ingrid Bispo, Emil Havránek, Friederike Aulenbacher, Alexandrina Cardoso, Kristýna Hrachovinová, Cristina Pinto, Selina Kim, Marc Lazarovici, The PROGRESSION Consortium

**Affiliations:** 1 RISE-Health, Faculty of Medicine, University of Porto, Porto, Portugal; 2 Faculty of Medicine, University of Porto, Porto, Portugal; 3 Department of Gynecology and Obstetrics, University Hospital Brno – Bohunice and Maternity Hospital, Brno, Czechia; 4 Faculty of Medicine, Masaryk University (MUNI MED), Brno, Czechia; 5 Katholische Stiftungshochschule, University of Applied Science, Munich, Germany; 6 RISE-Health, Nursing School, University of Porto, Porto, Portugal; 7 Institut für Notfallmedizin und Medizinmanagement (INM), LMU University Hospital, Munich, Germany; 8 LMU Munich, Munich, Germany

**Keywords:** labor, maternal positioning, mental models, midwifery students, multicenter study, pelvic biomechanics, virtual reality

## Abstract

**Introduction:**

Understanding maternal positioning and pelvic biomechanics is a core competency in midwifery education; however, conventional teaching methods provide limited opportunities to visualize internal anatomical changes during labor. The PROGRESSION project developed a virtual reality (VR)-enhanced educational program aimed at improving midwifery students’ knowledge and supporting immersive learning on the relationship between maternal positioning and labor progression. This study explored the educational effect, usability, and learner experience associated with the PROGRESSION VR training.

**Methods:**

A prospective, multicenter, pre–post intervention study was conducted between March and July 2025 across three midwifery education institutions in the Czech Republic, Germany, and Portugal. The intervention comprised a theoretical online module, a familiarization session, self-directed VR practice, and facilitated VR clinical scenarios. Knowledge of maternal positioning and pelvic biomechanics was assessed using a 10-item questionnaire administered before and immediately after the VR session. Usability was evaluated using the System Usability Scale (SUS) and the Virtual Reality System Usability Questionnaire (VRSUQ). Quantitative data were analyzed using nonparametric methods, and qualitative feedback was explored through thematic analysis.

**Results:**

Fifty-seven students completed both knowledge assessments and usability surveys. Although overall knowledge scores improved after the intervention, the change was not statistically significant (Sign test, *p* = 0.46). Only the Czech cohort demonstrated a significant knowledge increase (*p* = 0.02). SUS (mean 68 ± 13) and VRSUQ (mean 64 ± 13) scores indicated generally above-average usability, with no significant differences across institutions. Students reported high engagement, enhanced understanding of maternal positioning, and strong perceived educational value. Qualitative analysis identified three themes: functionality, visual clarity and realism, and educational value.

**Discussion:**

The PROGRESSION VR-enhanced training was well received and demonstrated high usability, offering an engaging and immersive learning experience that students perceived as educationally beneficial. Although knowledge gains were modest, the VR intervention was well accepted and perceived as a valuable complement to traditional instruction. The findings suggest that VR can enrich learning experiences, enhance students’ confidence in maternal positioning, and support comprehension of complex internal processes. Further research should explore repeated VR exposure, longitudinal curriculum integration, and assessment methods capable of capturing spatial and conceptual learning outcomes.

## 1 Introduction

Midwifery education is guided by international standards that emphasize competency in providing high-quality, woman-centered intrapartum care. The International Confederation of Midwives (ICM) and the World Health Organization (WHO) both highlight the importance of midwives supporting physiological labor and maternal choice in birthing positions (1, 2). Maternal positioning is recognized as a critical factor that positively influences pelvic mobility, fetal descent, and the physiological progression of labor (3–6). Accordingly, midwifery training frameworks worldwide incorporate core competencies in maternal positioning during labor, recognizing that appropriate positioning can facilitate labor progress and safe birth outcomes (1).

However, current midwifery curricula often rely heavily on didactic instruction, two-dimensional anatomical illustrations, or limited hands-on opportunities using mannequins or clinical placements. These conventional approaches often fall short in providing students with the spatial and dynamic understanding required to recognize how maternal positioning influences internal anatomical changes that affect fetal rotation and descent during labor. Because the most critical mechanisms of labor occur internally and cannot be observed directly, students frequently struggle to form accurate mental models of pelvic biomechanics, a challenge consistently identified in midwifery education (7, 8).

Simulation-based education (SBE) has increasingly been incorporated into midwifery training to address such limitations. Full-body mannequins, task trainers, and standardized patients are widely used to improve technical, cognitive, and communication skills while ensuring learner and patient safety (9, 10). Evidence demonstrates that SBE enhances clinical decision-making, increases learner confidence, and improves performance in obstetric emergencies (11, 12). Nevertheless, traditional simulation modalities often lack the capacity to accurately represent internal pelvic structures, fetal rotation and descent, or the biomechanical effects of various maternal positions (such as standing, squatting, hands-and-knees, or lateral postures), limiting students’ ability to visualize and internalize these dynamics. These persistent educational gaps underscore the need for more innovative training tools that offer intuitive and realistic representations of internal labor mechanics.

Virtual reality (VR) has emerged as a promising adjunct to SBE by providing immersive, interactive, three-dimensional visualizations of complex internal processes. In health professions education, VR has been shown to improve knowledge acquisition, spatial reasoning, skill performance, and learning efficiency compared with conventional methods alone (13, 14). Recent research in midwifery education suggests that VR can increase learner engagement and improve understanding of fetal descent, rotation, and maternal pelvic dynamics, thereby supporting a more intuitive grasp of labor physiology (15–19). Related work by Liu et al. (20) further highlights the potential and challenges of VR integration in obstetrics training, particularly in terms of balancing learning gains with user acceptability in childbirth delivery simulations.

The PROGRESSION project^1^, funded by the Erasmus+ program, responds to these educational needs by developing an innovative VR-based learning environment designed to enhance midwifery students’ understanding of maternal positioning and its biomechanical effects on labor. Specifically, the project has developed a VR-enhanced training program that enables students to visualize and practice positioning maneuvers while observing the resulting subtle movements of internal anatomical structures. Grounded in clinical case scenarios, the VR tool allows learners to actively position the pregnant woman and observe how specific maternal positions (such as standing, squatting, hands-and-knees, or lateral) affect pelvic inlet and outlet diameters and fetal rotation and descent. By integrating immersive technology with evidence-based theoretical content, PROGRESSION aims to enhance students’ anatomical knowledge, spatial reasoning, and clinical decision-making, ultimately strengthening their readiness to support physiological labor.

This exploratory study primarily aims to evaluate the educational effect of the PROGRESSION VR-enhanced training program on midwifery students’ knowledge of maternal positioning and pelvic biomechanics. A secondary objective is to assess the usability of the VR application and explore students’ perceptions of the overall learning experience.

## 2 Materials and methods

### 2.1 Study design

This prospective, international, multicenter pre–post intervention study was conducted at three higher education institutions delivering midwifery education: the *Faculty of Medicine at Masaryk University* (Czech Republic), *Katholische Stiftungshochschule München* (Germany), and the *Nursing School of Porto* (Portugal). The study was implemented between March and July 2025, and all learning activities were integrated into existing curricula. No control group was used, as the primary aim was to evaluate changes in knowledge following exposure to the PROGRESSION educational program. Ethical approval was obtained from the institutional ethics committees of all participating institutions prior to data collection, and all study procedures were conducted in accordance with the Declaration of Helsinki. A study information sheet in the participants’ native language, detailing all ethical aspects and data-protection measures, was provided prior to the electronic signing of informed consent. All participants were assured of anonymity and voluntary participation.

### 2.2 Recruitment and eligibility criteria

Eligible participants were midwifery students enrolled at the participating institutions and aged 18 years or older. To ensure comparable baseline knowledge within each educational system, participants were recruited at program-specific stages that correspond to similar curricular progression in maternal and intrapartum care. In Portugal, where midwifery education is exclusively postgraduate, students were enrolled in the second semester of the first year, whereas in Germany and the Czech Republic, where midwifery is an undergraduate program, students were enrolled in the second semester of the second year. Students reporting medical conditions incompatible with safe VR use (e.g., vestibular or seizure disorders) were excluded. Recruitment was conducted within scheduled course activities. Participation was voluntary and had no impact on academic formal assessment.

### 2.3 Experience setup

All sessions were conducted in a controlled environment designed to ensure participant safety and consistency across study sites. At least one trained researcher or facilitator was present throughout each intervention session (including the familiarization period, VR training, and knowledge assessments) to monitor participants, ensure correct use of the equipment, and provide procedural guidance when necessary.

**Figure 1:**
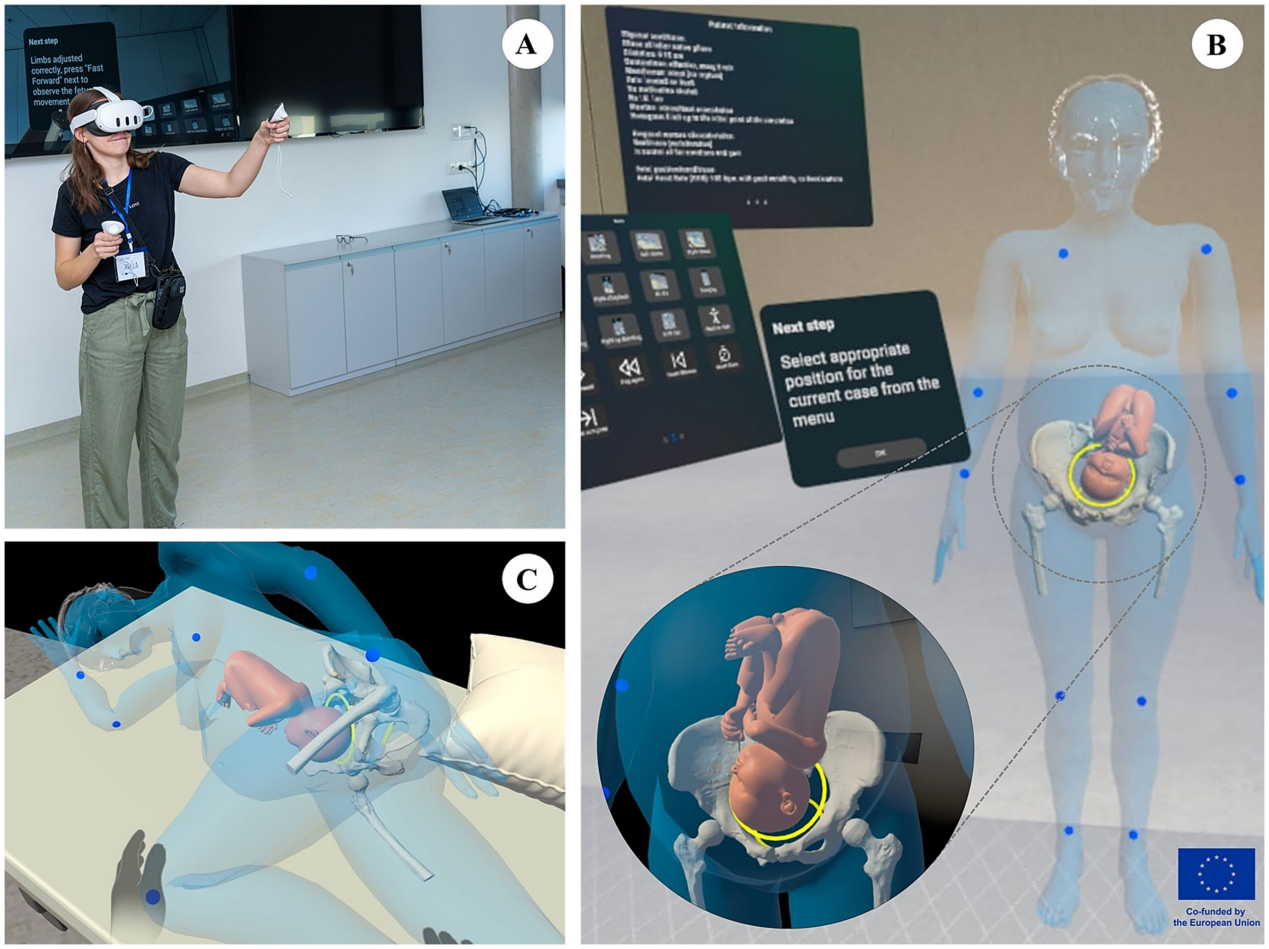
PROGRESSION study images showing the study setting **(A)** and key app features **(B,C)**.

VR activities took place in a dedicated room that was sufficiently large to allow free movement and to ensure a safe and controlled learning environment. The room was arranged to maintain participant privacy and to prevent interruptions during VR use, thereby minimizing potential distraction or startle responses while wearing the headset. Rooms were selected to reduce physical hazards, such as excessive furniture, sharp corners, or narrow spaces (Figure 1A).

Each site used Meta Quest 3 headsets. All devices were fully charged before each session, and the VR application was pre-installed. A computer paired with the headsets and connected to a large monitor, was used to mirror the VR experience for facilitators and other students, enabling real-time observation and guidance. After each use, headset interfaces were sanitized in accordance with local infection prevention protocols.

The developed VR application featured a full-size three-dimensional model of a pregnant woman with partially transparent anatomical layers, enabling visualization of internal structures (Figure 1B), including the maternal pelvis and fetus (Figure 1B, zoomed circle). Maternal joints could be manipulated using the controller or pinch-gesture functionality, allowing users to adjust the model to achieve specific maternal positions (Figure 1C). Additional interface elements included a scenario-selection menu, clinical case descriptions, controls for scenario progression, and context-sensitive informational pop-ups designed to support decision-making and enhance learning (Figure 1B, upper left). The VR application provided identical content and interface features across all study sites, ensuring consistency in the educational experience delivered to participants.

### 2.4 Data collection instruments

#### 2.4.1 Sociodemographic data

Sociodemographic data, including age, gender, academic background, and previous experience with simulation-based training or VR systems, were collected through an online survey administered prior to study enrollment.

#### 2.4.2 Knowledge assessment

Students’ knowledge was evaluated using a 10-item, multiple-choice, clinical case–based online questionnaire administered at baseline and immediately after the VR intervention. The items reflected the core educational content of the PROGRESSION program, including pelvic anatomy, biomechanics of labor, and decision-making regarding maternal positioning. Item development followed a consensus-based process involving midwifery and obstetric experts from all partner institutions. Each item was scored as correct or incorrect, and total scores were expressed as percentages (0–100). The instrument was designed for within-subject pre–post comparison and did not undergo formal psychometric validation. To ensure linguistic clarity and conceptual equivalence, the questionnaire was translated into the native language of each participating country and cross-checked by bilingual faculty members. The English version is provided as Supplementary material.

#### 2.4.3 Usability assessment

Usability of the VR application was assessed using two validated instruments: the System Usability Scale (SUS) (21) and the Virtual Reality System Usability Questionnaire (VRSUQ) (22).

SUS is a widely used 10-item measure of perceived usability, scored on a 5-point Likert scale. Items alternate between positive and negative phrasing. The total SUS score is calculated using the following standard procedure:

- For odd-numbered items: score = response – 1.
- For even-numbered items: score = 5 – response.
- SUS total score = (sum of adjusted scores) × 2.5.

This yields a score ranging from 0 to 100. Scores >67 are generally interpreted as indicating above-average usability.

VRSUQ is a recently developed VR-specific usability instrument comprising 9 items rated on a 5-point Likert scale, with items 3, 8, and 9 reverse-coded. Items are grouped into three usability domains: Effectiveness (items 1 to 3), Efficiency (items 4 to 6), and Satisfaction (items 7 to 9). The final score is computed as:

- VRSUQ score = (mean of adjusted item scores − 1) × 100/4.

This results in a 0–100 scale analogous to SUS. A score of >67 similarly reflects above-average usability.

Both instruments were administered in English to preserve the validity of the original scales.

#### 2.4.4 Overall experience assessment

Approximately two to 4 weeks after the VR-enhanced learning experience, participants completed an online follow-up questionnaire designed to capture their overall perception of the educational program. This instrument assessed aspects such as the integration of the different teaching modalities, perceived confidence in applying maternal positioning techniques, clarity and appropriateness of the learning content, level of interactivity and engagement, the perceived relevance of the VR scenarios to real clinical situations, and the usefulness of the feedback provided during the facilitated sessions. The instrument included both Likert-scale items and open-ended questions to capture quantitative ratings and qualitative insights.

### 2.5 Study protocol

**Figure 2:**
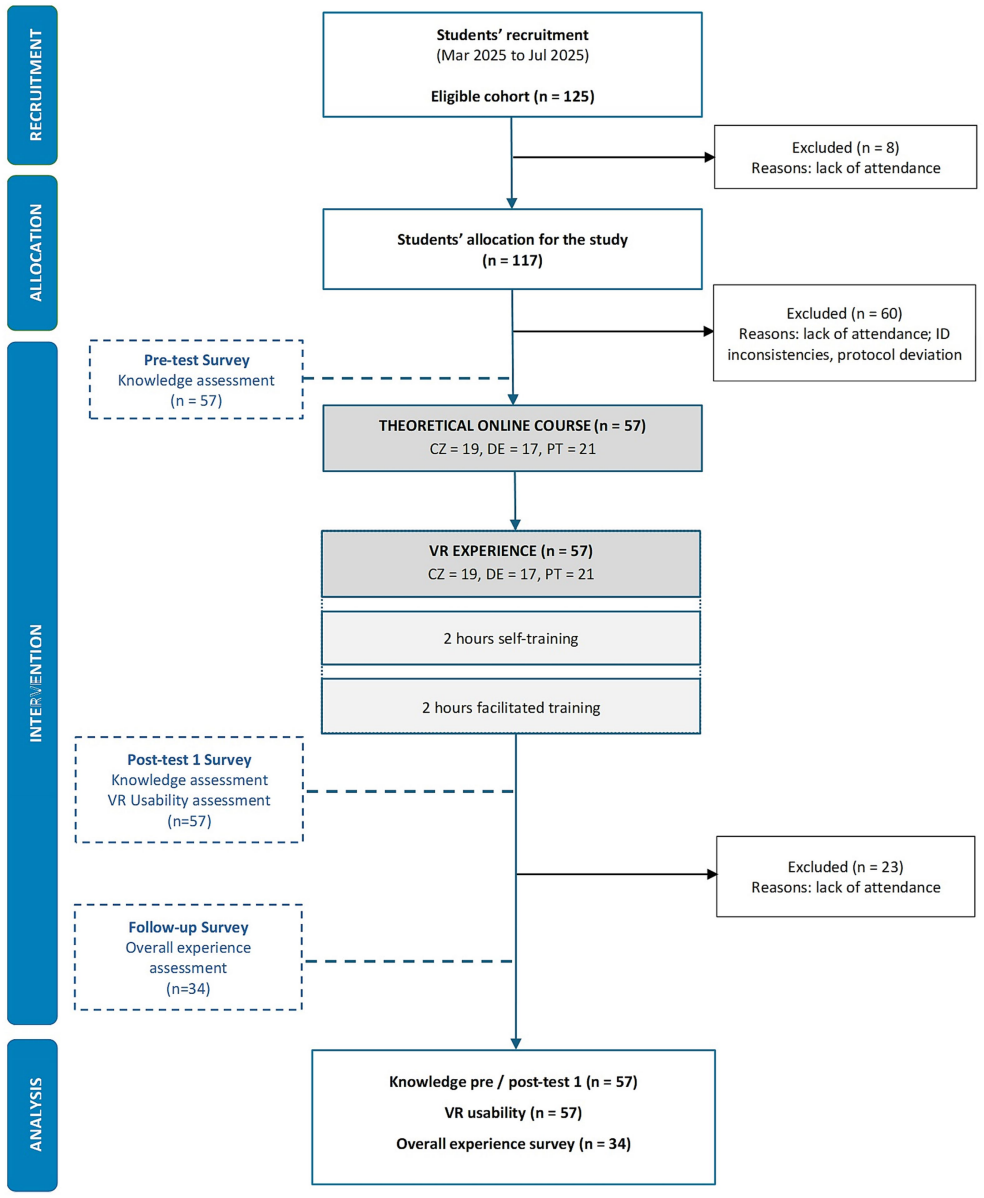
Study flow diagram.

A standardized protocol and facilitator manual were implemented to ensure consistency across all study sites, and the overall study flow is presented in Figure 2. The protocol comprised five sequential phases.

#### 2.5.1 Pre-test

Participants completed an online questionnaire assessing their knowledge of optimal maternal positioning and clinical decision-making during labor. Responses were recorded using unique individual codes to enable paired comparisons while preserving anonymity.

#### 2.5.2 Theoretical online module

Students engaged in an asynchronous theoretical module delivered through an online learning platform. The module addressed pelvic anatomy, biomechanics of labor, and maternal positions designed to facilitate fetal descent. Each section concluded with a formative quiz, and a score of 100% was required to progress to the next section. Unlimited attempts were permitted. The module content was identical across institutions and was translated into the native language of each participating country to ensure linguistic clarity.

#### 2.5.3 VR-based educational experience

Approximately two to 4 weeks after completing the online module, students attended an in-person session during which they first participated in a structured familiarization period. Small groups of students were introduced to the VR equipment and environment, and each participant spent about 30 min exploring the system to increase comfort and reduce the likelihood of cybersickness during the formal training.

The VR-based intervention was delivered to groups of four students accompanied by one trained facilitator and consisted of two components:

1. The self-directed training component lasted 2 hours, during which students explored basic maternal positioning techniques, including positions that illustrate pelvic nutation and counternutation. Each student interacted with the VR environment for approximately 30 min while the remaining time was used to observe peers.
2. The facilitated training component also lasted 2 hours and involved guided participation in three immersive clinical scenarios representing lack of fetal engagement, arrested labor, and fetal malposition. Each student actively completed one scenario for approximately 15 min with real-time guidance from the facilitator and subsequently received 10–15 min of individualized feedback. During each scenario, the other students and the facilitator observed the session via screen mirroring, allowing for shared learning and group discussion.

#### 2.5.4 Post-test

Immediately after the VR experience, students completed the same online knowledge questionnaire administered at baseline, with the order of questions and response options randomized to minimize recall bias. They also completed the two usability surveys (SUS and VRSUQ).

#### 2.5.5 Follow-up

Approximately two to 2 weeks after the VR-enhanced learning experience, participants completed an online follow-up questionnaire evaluating their overall experience with the intervention.

### 2.6 Statistical analysis

No formal sample size calculation was performed, considering the exploratory nature of the study and the intention to include the full cohort of students enrolled in the participating midwifery programs. Comparative analyses were conducted on complete cases. Categorical variables were compared using the chi-square test, with Fisher’s exact test applied when expected cell counts were <5. The distribution of continuous variables was assessed using the Kolmogorov–Smirnov test, supported by visual inspection of histograms and Q–Q plots, which indicated non-normality. Consequently, comparisons between baseline and post-intervention knowledge scores were conducted using the non-parametric Sign test (overall and stratified by institution). Between-institution differences in knowledge change scores were evaluated with the Kruskal–Wallis test. Usability scores obtained from the SUS and VRSUQ surveys were summarized descriptively, and between-institution comparisons of usability scores were performed using the Kruskal–Wallis test. Open-ended responses from the overall experience questionnaire were analyzed using thematic analysis to identify recurring themes and provide additional insights into students’ experiences with the intervention. All statistical analyses were performed using IBM SPSS Statistics, version 29.0.2.0, and a two-tailed significance level of *p* < 0.05 was applied for inferential analyses.

## 3 Results

### 3.1 Participant enrolment and sociodemographic characteristics

A total of 125 midwifery students comprised the eligible cohort across the three partner institutions. Of these, 57 students completed both the pre- and post- intervention knowledge assessments as well as the usability surveys, and 34 completed the follow-up survey (Figure 2).

Table 1 summarizes the sociodemographic characteristics of the sample and between-country comparisons. Following attrition, 17 students (29.8%) from Germany, 19 (33.3%) from the Czech Republic, and 21 (36.8%) from Portugal constituted the final analytic sample. The mean age was 24.4 years, and the sample was predominantly female (98.2%), reflecting the gender distribution typical of midwifery programs. Gender distribution was homogeneous across sites, with almost all participants identifying as female. A significant difference in age was observed among institutions (Kruskal–Wallis test, *p* < 0.001), with Portuguese students being markedly older than those from Germany and the Czech Republic, consistent with program-specific demographic differences.

**Table 1:** Sociodemographic data of the participants included in the study.

Sociodemographic variables	Overall (*n* = 57)	Czech Republic (*n* = 19)	Germany (*n* = 17)	Portugal (*n* = 21)	*p*-value*
Age					
Mean ± SD	24.4 ± 4.5	21.6 ± 1.6	22.7 ± 3.2	28.4 ± 4.4	**<0.001**
Gender *n* (%)					
Female	56 (98.2%)	19 (100%)	16 (94.1%)	21 (100%)	—
Male	1 (1.8%)	—	1 (5.9%)	—	
Previous experience with simulation *n* (%)					
Yes	30 (52.6%)	14 (73.7%)	11 (64.7%)	5 (23.8%)	**0.003**
No	27 (47.4%)	5 (26.3%)	6 (35.3%)	16 (76.2%)	
Previous experience with VR technology *n* (%)					
Yes	15 (26.3%)	4 (21.1%)	2 (11.8%)	9 (42.9%)	0.078
No	42 (73.7%)	15 (78.9%)	15 (88.2%)	12 (57.1%)	

*n*: number of participants; SD, standard deviation; VR, virtual reality.

*Statistical tests: age: Kruskal–Wallis test. Simulation experience and VR experience: Chi-square tests. Gender distribution was not tested due to near-complete homogeneity. Bold indicates statistical significance.

Regarding previous exposure to educational technologies, nearly half of the students (47.4%) reported no prior experience with simulation-based learning, and a substantial majority (73.7%) indicated no previous use of VR technology. These findings highlight the exploratory nature of introducing a novel VR-enhanced training intervention in this educational context. Significant differences were found in previous exposure to simulation-based learning (*χ*^2^ test, *p* = 0.003), with Portuguese students reporting markedly less simulation experience compared with their German and Czech counterparts. Differences in prior experience with VR technology did not reach statistical significance (*χ*^2^ test, *p* = 0.078), although descriptively, Portuguese students reported higher exposure to VR than students from the other institutions.

### 3.2 Knowledge assessment outcomes

**Figure 3:**
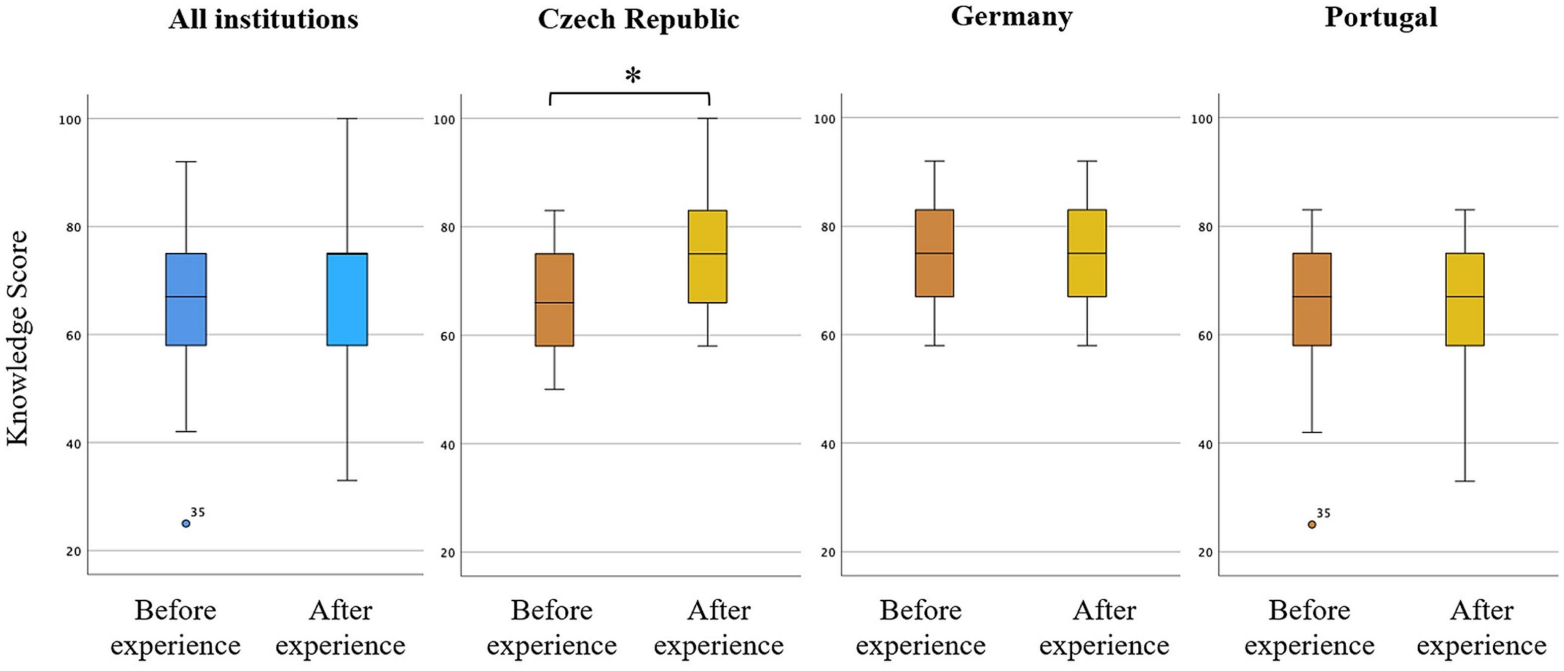
Box-plots of the knowledge scores before and after the educational experience, for the full sample and stratified by country. Scores (0–100). Statistically significant difference marked with (*).

Table 2 and Figure 3 present the changes in students’ knowledge scores from pre-test to post-test. Across the full sample, median knowledge scores increased following the VR-enhanced educational experience, although this difference was not statistically significant (Sign test, *p* = 0.46). When analyzed by institutions, however, students from the Czech Republic demonstrated a statistically significant increase in knowledge scores (Sign test, *p* = 0.02), while no statistically significant differences were observed in the other participating institutions. Comparison of knowledge score changes across institutions revealed no significant differences in the magnitude of improvement between sites (Kruskal–Wallis test, *p* = 0.168).

**Table 2:** Knowledge assessment scores before and after the educational experience.

Knowledge level median (IQR)	All (*n* = 57)	Czech Republic (*n* = 19)	Germany (*n* = 17)	Portugal (*n* = 21)
Baseline (pre-test)	67 (17)	66 (17)	75 (16)	67 (17)
After VR experience (post-test)	75 (17)	75 (17)	75 (16)	67 (21)
*p*-value*	0.46	**0.02**	1.00	0.82

Values in %. Bold value indicates statistical significant.

VR, virtual reality; *n*, number of participants. *Sign test.

### 3.3 Usability outcomes

Usability outcomes of the VR application are presented in Table 3. Across the full sample, the VR application achieved a mean SUS score of 68 ± 13, indicating overall above-average usability. VRSUQ total usability scores presented slightly lower values (mean 64 ± 13). Usability perceptions varied across institutions. Students from the Czech Republic (69 ± 12) and Portugal (70 ± 17) reported SUS scores that met or exceeded the above-average usability threshold, whereas German students reported lower SUS scores (64 ± 8), falling below this benchmark. A similar pattern emerged for VRSUQ scores, where Germany again reported the lowest usability (54 ± 8) compared with the Czech Republic (68 ± 9) and Portugal (67 ± 17). However, a Kruskal–Wallis test indicated that these differences were not statistically significant for either SUS (*p* = 0.403) or VRSUQ (*p* = 0.139) scores.

**Table 3:** Usability scores (mean ± SD) of virtual reality educational experience (*n* = 57).

Participant cohort (*n*)	SUS	VRSUQ
Czech Republic (*n* = 19)	69 ± 12	68 ± 9
Germany (*n* = 17)	64 ± 8	54 ± 8
Portugal (*n* = 21)	70 ± 17	67 ± 17
All (*n* = 57)	68 ± 13	64 ± 13

Scores (0–100).

*n*, number of participants; SD, standard deviation; SUS, system usability scale; VRSUQ, virtual reality system usability questionnaire.

Analysis of the VRSUQ subdomains (effectiveness, efficiency, and satisfaction, rated on a 1–5 scale) further supported this pattern. Across the full sample, efficiency received the highest ratings (3.9 ± 0.9), followed by satisfaction (3.5 ± 1.4) and effectiveness (3.3 ± 1.2). German students reported lower usability across all dimensions compared with their Czech and Portuguese counterparts (Table 4).

**Table 4:** Mean ± SD scores for the VRSUQ usability dimensions (*n* = 57), rated on a 1–5 scale.

Participant cohort (*n*)	Effectiveness	Efficiency	Satisfaction
Czech Republic (*n* = 19)	3.4 ± 1.0	4.0 ± 0.8	3.8 ± 1.0
Germany (*n* = 17)	3.1 ± 1.0	3.6 ± 0.9	2.8 ± 1.5
Portugal (*n* = 21)	3.3 ± 1.4	4.1 ± 1.0	3.7 ± 1.4
All (*n* = 57)	3.3 ± 1.2	3.9 ± 0.9	3.5 ± 1.4

*n*, number of participants; SD, standard deviation; VRSUQ, virtual reality system usability questionnaire.

### 3.4 Feedback on the overall experience

Overall feedback on the educational intervention indicated a positive learner experience across multiple components of the program. The combination of various teaching methods (self-learning module, VR activities, and facilitator feedback) was perceived as enhancing students’ learning experience (3.9 ± 1.1, scale 1–5). Students also reported greater confidence in applying maternal positioning techniques following the training (3.9 ± 1.0) and agreed that the learning content effectively supported their understanding of key concepts (3.8 ± 1.1). The difficulty level of the program was viewed as appropriate for their skills and knowledge (3.8 ± 1.0). Feedback on the VR component was similarly favorable. Participants described the VR experience as interactive and engaging (3.9 ± 1.1) and indicated that VR practice sessions improved their ability to adapt maternal positioning in real clinical situations (3.9 ± 1.1) Facilitated debriefing following the VR activities was also rated high, with students recognizing the value of the constructive and supportive feedback (3.9 ± 1.2).

Most students endorsed the VR application as a valuable educational tool, with 31 students (91%) indicating that the VR-based program could be useful for midwifery education. Only one participant (3%) responded negatively, and two participants (6%) indicated it might be somewhat useful.

Analysis of open-ended responses further reinforced the positive perception of the VR-enhanced educational experience. Three overarching themes were identified: functionality, visual clarity and realism, and educational value (Table 5). Under the theme of functionality, students described the VR system as intuitive and easy to use. The theme of visual clarity and realism highlighted the perceived benefits of enhanced visualization of maternal positioning, fetal orientation, and pelvic anatomy. Finally, under the theme of educational value, students emphasized that VR supported their understanding of clinical scenarios and facilitated a deeper comprehension of maternal positioning strategies. Representative student quotes are presented in Table 5.

**Table 5:** Thematic analysis of the open questions in the overall experience survey.

Identified themes		Students’ representative quotes
Functionality	Students consistently noted VR as easy to use and intuitive.	“It was easy to use.” “Easy to handle.”
Visual clarity and realism	VR allowed clearer perception of maternal/fetal positions, pelvic anatomy, and spatial awareness.	“VR mode is better for visualization and understanding what’s happening.” “Better visualization of the fetal position.”
Educational value and understanding	VR was perceived as helpful for comprehending clinical scenarios and learning maternal positioning.	“It’s better for understanding clinical cases.” “It was helpful to see what is happening to the woman.” “You can better understand the maternal positions.”

## 4 Discussion

The PROGRESSION project aimed to respond to a well-recognized gap in midwifery education: the limited practical training dedicated to maternal positioning during labor. By incorporating a VR-enhanced learning program, the project sought to strengthen students’ understanding of internal anatomical structures and how these structures interact dynamically during labor.

### 4.1 Educational impact

Although students showed higher scores in the post-test compared with the pre-test, the improvement was not statistically significant across the full sample. This pattern is consistent with the broader VR education literature, in which single-session or short-term interventions often produce limited knowledge gains that may not reach statistical significance. Kyaw et al. (14) reported that VR can improve knowledge acquisition, but effect sizes tend to be small and closely dependent on exposure duration and instructional depth. Similarly, Chen et al. (23) found moderate knowledge gains among nursing students. These findings parallel our results, suggesting that a single VR exposure may be insufficient to generate measurable changes in theoretical knowledge assessed immediately after the intervention (24).

Additionally, the complexity of the educational content (maternal positioning, pelvic biomechanics, and fetal rotation) may require repeated practice and reinforcement. While VR fosters spatial reasoning and internal visualization, these benefits may require repeated exposure, as a single VR session may not always translate into measurable knowledge gains. Prior obstetric VR studies similarly reported no significant improvement in written knowledge assessments despite high learner engagement and perceived benefit (17, 19), suggesting that the educational gains facilitated by immersive environments may primarily occur in cognitive domains not measured by standard recall-based tests.

In the present study, the multicenter and international nature of the design limited the types of assessments that could be implemented. Variability in institutional resources required a standardized and feasible testing method across sites, leading to the selection of case-based multiple-choice questions. Although this format offers consistency, it primarily assesses factual recall and may inadequately reflect the higher-order cognitive processes (such as situational understanding, clinical decision-making, and the development of mental models) that VR is designed to foster. Research in simulation-based learning (25, 26) highlights that traditional tests often fail to detect growth in conceptual reasoning or applied problem-solving that emerges during experiential activities. A literature review on immersive VR (13) similarly concluded that commonly used outcome measures are often misaligned with the pedagogical benefits offered by the technology. Thus, the modest gains observed may represent a mismatch between assessment tools and the true nature of learning outcomes, rather than an absence of educational benefit. Importantly, many participants anecdotally reported that the VR experience improved their understanding of pelvic dynamics and fetal positioning. Such improvements in conceptual and spatial understanding are a recognized strength of VR-based education (27), although they may not be evident in traditional multiple-choice assessments.

Considering that the intervention aimed to be adopted in diverse midwifery programs, the educational program combined a theoretical self-directed module with a VR-enhanced practical component followed by facilitated reflection. This multimodal design was intentional, grounded in simulation best practices that emphasize the importance of structured preparation and debriefing as essential components of experiential learning (28–30). This design likely allowed the VR to act as an adjuvant, reinforcing concepts introduced during the theoretical phase and supporting the development of more robust mental models. This synergistic effect is consistent with evidence showing that VR is most effective when used as a complementary tool that enhances visualization, learner engagement, and conceptual understanding (14, 18).

The institution-specific analysis revealed that only students from the Czech Republic showed a statistically significant increase in knowledge scores. This suggests that contextual or procedural differences may have influenced learning outcomes, such as variations in curricular structure, group dynamics, and local teaching context. Another contributing factor could be the facilitator’s role during implementation. Despite adherence to a standardized protocol, variations in the degree of support, depth of explanations, or the manner in which reflection and discussion were guided may have contributed to inter-site variability. These findings underscore the importance of consistent facilitator preparation in educational interventions involving immersive technologies. Additional factors may have contributed to this site-specific finding. Baseline scores differed across cohorts, with the German cohort starting at a higher median level, which may have limited measurable gains due to a potential ceiling effect. Differences in learner characteristics (e.g., age distribution and prior exposure to simulation-based learning or VR) may also have influenced familiarity with immersive learning and responsiveness to the intervention. Across cohorts, pre- and post-test interquartile ranges were broadly comparable, suggesting similar dispersion in performance. Where post-test variability appeared slightly wider, this may reflect heterogeneous individual learning responses rather than a uniform effect, and should be interpreted cautiously given the exploratory sample size.

### 4.2 Usability and learner experience

The strong positive reception of the PROGRESSION VR application is an encouraging sign for its future use. Both usability instruments indicated that students found the system generally easy to use, engaging, and effective as a learning aid. SUS scores averaged ≈68, exceeding the benchmark for acceptable usability (21). The VR-specific usability questionnaire (VRSUQ) further corroborated positive user experience, though some variability across sites was noted. The observed differences could be due to the implementation across three countries (e.g., varying technical support), or the fact that nearly half of our participants were completely new to VR. In fact, the German cohort, which gave the lowest SUS (≈64), had among the highest prior simulation exposure (perhaps setting higher expectations) but lowest prior VR exposure; conversely, the Portuguese cohort, despite less simulation background, had somewhat more VR familiarity and rated usability higher. These results reflect trends observed across VR applications in health professions education, where usability is typically rated positively even when technical performance varies (23). Systematic reviews highlight that immersive technologies frequently receive favorable user evaluations due to their interactive nature, novelty, and ability to foster learner motivation and engagement (24).

Beyond usability metrics, the learning experience itself was rated positively. Most participants described the VR tool as enjoyable, user-friendly, and educationally valuable. These perceptions mirror results from VR studies in other fields (31–34), where learners consistently report high satisfaction and view VR as a meaningful enhancement to conventional teaching approaches. The majority agreed that the combination of methods (self-directed theoretical module, hands-on VR practice, and facilitated debriefing) enriched their learning. Participants in our study specifically valued the real-time guidance and group discussion during the facilitated scenarios, noting that it helped them link the virtual experience to real clinical reasoning. Particularly relevant is the appreciation for internal visualization, as students highlighted VR’s capacity to clarify maternal and fetal relationships, pelvic anatomy, and spatial dynamics. Students nearly universally endorsed VR as a worthwhile addition to midwifery curricula. This corresponds with prior reports where learners not only enjoyed VR but also recommended its broader adoption in training programs (35). When applied to support spatial reasoning and the visualization of internal anatomy, VR tends to be well accepted and positively perceived by learners (17, 19). In contrast, when used as a standalone modality for hands-on skill training, VR may yield learning improvements but also elicit more variable acceptance and contextual feasibility (20). These contrasts highlight the need to align educational technologies with the intended learning outcomes, whether focused on conceptual understanding, clinical decision-making, or manual competencies (28, 29).

As observed in other VR studies, a minority of participants reported physical discomfort, including dizziness and unpleasant sensations. By providing an initial orientation and allowing students to explore the virtual environment for ~30 min beforehand, we aimed to mitigate adverse effects like cybersickness, and these mild symptoms did not significantly impact overall engagement or satisfaction reported by the students.

### 4.3 Relevance for midwifery education

Our findings contribute to a growing body of literature exploring how virtual and immersive simulations can augment midwifery education. Until recently, the application of VR in midwifery training has lagged behind its uptake in some other health disciplines, but evidence is rapidly emerging to support its benefits. Several recent studies (15, 17, 19, 27) echo our key observations.

When situating our VR intervention among similar innovations, a few unique strengths and differences emerge. One strength was the multicenter design encompassing different education systems (undergraduate and postgraduate) and cultural contexts. To our knowledge, this is one of the first evaluations of VR in midwifery education that spans multiple countries. Despite variations in curricula and student profiles, we observed broadly consistent patterns in how the VR training was received, indicating a degree of generalizability. In all locations, students with minimal prior VR exposure were able to use the technology effectively and found it beneficial.

Another important dimension is how VR training might influence attitudes and future practice. A core aim of our intervention was to reinforce the value of supporting physiological birth positions, which is a competency emphasized by international standards (1, 2). Despite clear evidence that upright or lateral positions can shorten labor and reduce complications (3–5), many maternity settings still default to supine positioning. Education can be a powerful tool to change this paradigm. By allowing students to witness the anatomical rationale for upright positions (such as increased pelvic diameters or enhanced fetal rotation) in the VR scenarios, we aimed to strengthen their commitment to evidence-based practice. The fact that students’ confidence in adapting maternal positions increased after the training is promising. It suggests they are more likely to advocate for and implement these practices in clinical settings. This outcome aligns with the findings of Lin et al. (16), where midwives trained with a birthing positions simulation subsequently achieved a higher rate of non-supine births in their unit.

In terms of practical challenges, our experience highlighted several factors that educators and institutions should plan for when integrating VR. One is the learning curve, not just for students, but also for faculty facilitators. We invested time in training facilitators to use the VR system and guide scenarios, which was essential to the smooth running of sessions. Participants benefited from having someone present who could troubleshoot or provide cues, underscoring that VR is most effective as part of a well-facilitated session rather than a standalone tool. This finding echoes the recommendation by Saab et al. (35) that VR simulation be “underpinned by a strong pedagogy” and accompanied by proper training of educators.

### 4.4 Limitations

This study has several limitations that should be considered when interpreting the findings. First, the relatively small sample size limits statistical power and reduces generalizability, especially for cross-site comparisons. Second, we lacked a control group or comparison condition. Without a non-VR control, we cannot definitively attribute the observed knowledge gains to the VR intervention itself. Third, recruitment and procedural issues (such as incorrect participant identifiers and deviations from the study protocol) may have introduced variability across institutions. Fourth, the knowledge assessment we used was a brief 10-item questionnaire. While it was tailored to the learning objectives, its sensitivity and scope were limited. More comprehensive testing or skill-based assessments (e.g., OSCE stations, objective measurements of positioning technique) might reveal learning gains that our questionnaire could not detect. Additionally, the lack of a statistical difference between pre- and post-test knowledge scores may partly reflect test–retest bias, as students completed the same questionnaire at two different time points. Differences in formal educational level across institutions may also have influenced baseline knowledge and learning outcomes; although cohorts were selected at comparable curricular stages and analyses were stratified by institution, residual confounding cannot be excluded. Finally, our study only assessed immediate post-intervention knowledge; we did not examine long-term knowledge retention or skill performance in clinical settings. This short follow-up means we do not know whether the modest learning gains and high enthusiasm translate into lasting educational benefits or improved clinical competencies. Future studies should include larger samples and incorporate in-depth qualitative methods (e.g., interviews or focus groups) as well as controlled designs to better understand learning mechanisms and contextual factors influencing VR implementation.

## 5 Conclusion

The PROGRESSION project demonstrates the valuable role that immersive technologies can play in strengthening midwifery education, particularly in areas that require deep understanding of internal anatomical relationships and dynamic labor processes. As an exploratory study, it highlights both the educational benefits and the practical challenges involved in integrating VR into routine teaching. Future work should explore repeated and longitudinal VR exposure, ideally with larger controlled trials or longitudinal designs, to evaluate knowledge retention and skill transfer to clinical practice. In addition, future studies should adopt assessment methods capable of capturing the spatial–conceptual learning that VR is particularly well suited to support. Overall, the findings encourage continued development and broader implementation of VR-enhanced educational approaches in midwifery and other health professions.

## Data Availability

The datasets presented in this article are not readily available because they are subject to institutional data-sharing policies. Requests to access the datasets should be directed to Carla Sa-Couto, csacouto@med.up.pt.

## Footnotes

1
https://progression-project.eu/

## The PROGRESSION consortium

Abel Nicolau, RISE-Health, Faculty of Medicine, University of Porto (FMUP), Porto, Portugal. Arminda Nunes, RISE-Health, Nursing School, University of Porto, Porto, Portugal. Carla Cerqueira, RISE-Health, Nursing School, University of Porto, Porto, Portugal. Dominika Králová, Department of Health Sciences, Faculty of Medicine, Masaryk University; Department of Gynecology and Obstetrics, University Hospital Brno – Bohunice and Maternity Hospital, Masaryk University (MUNI MED), Brno, Czech Republic. Frieder Pankratz, Institut für Notfallmedizin und Medizinmanagement (INM), LMU University Hospital, Munich, Germany. Jennifer Tempfli, Institut für Notfallmedizin und Medizinmanagement (INM), LMU University Hospital, Munich, Germany. Lukáš Hruban, Department of Gynecology and Obstetrics, University Hospital Brno – Bohunice and Maternity Hospital, Department of Health Sciences – Departments of Non-Medical Branches, Faculty of Medicine, Masaryk University (MUNI MED), Brno, Czech Republic. Marika Bajerová, Rehabilitation Clinic, University Hospital Brno – Bohunice and Maternity Hospital, Department of Physiotherapy and Rehabilitation – Departments of Non-medical Branches, Faculty of Medicine, Masaryk University (MUNI MED), Brno, Czech Republic. Matěj Anton, Department of Gynecology and Obstetrics, University Hospital Brno – Bohunice and Maternity Hospital, Faculty of Medicine, Masaryk University (MUNI MED), Brno, Czech Republic.
