# Supplementary material for: Impact of a virtual reality-enhanced learning program for maternal positioning in midwifery students: an exploratory multicenter pre–post study
Source: Front Med (Lausanne). 2026 Mar 6;13:1771624. doi: 10.3389/fmed.2026.1771624 (PMC13002410; doi:10.3389/fmed.2026.1771624)
Supplement: Supplementary file 1 [file Data_Sheet_1.PDF]

KNOWLEDGE TESTING (MCQs)

1. What is the incorrect midwifery intervention for positioning of the patient during a passive 2nd stage of labour? **(NO Scenario)**

a) To facilitate nutation.

b) To promote vertical integration of the sacrum by forward movement and the lowering of the tip of the coccyx, thus reduction of the pelvic outlet. INCORRECT – COUNTERNUTATION

c) To encourage patient to move to modified semi-prone position (exaggerated Sim´s position).

d) To increase of the pelvic outlet.

e) Forward movement and the lowering of the promontory, thus reduction of the pelvic inlet.

2. Choose the proper sequence for the favorable rotation in case of fetal occiput is placed over right sacroiliac joint. **(Occiput Posterior Scenario)**

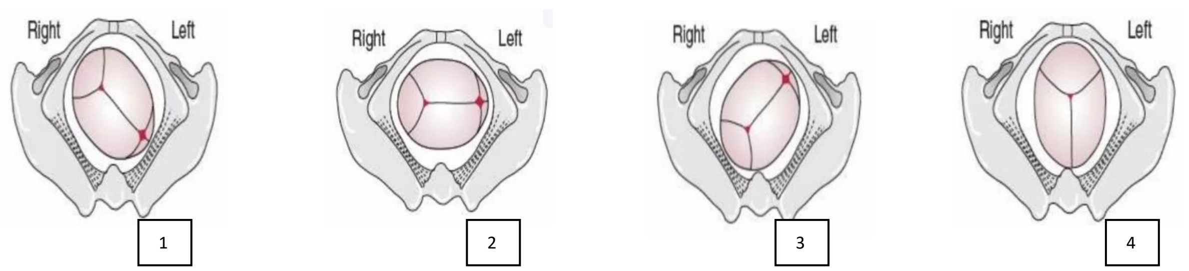

a) 1, 2, 3, 4

b) 3, 1, 2, 4

c) 3, 4, 1, 2

d) 3, 2, 1, 4

e) 3, 2, 4, 1

3. Julia is in active phase of labour. She is recumbent lateral position at her left side. She is complaining of mild to severe back pain and exhaustion. No medication required. No I.V. line sited. Fetal heart beat (FHR) is reassuring per intermittent auscultation.

- Vaginal findings: Dilatation: 8-10 cm.
- Membranes: intact (not ruptured).
- Contractions: effective, every 3 min.

What is the midwifery suggestion for action? **(ROP Scenario)**

a) Modified Lateral Recumbent Position w/ or w/o rocking motion.

b) Supported Hands-and-knees "IN" position “All 4´s”.

c) Supported Full Deep Squat.

d) Walcher's.

e) Standing.

4. If the midwife finds out the “flattened” area of fetal skull, anterior fontanelle is easily felt during vaginal examination so there is the concern of malposition. The correct action plan for positioning the patient is to allow her to common recumbent position? **(NO Scenario)**

a) True

b) False

5. See the picture bellow and answer the following questions. **(NO Scenario)**

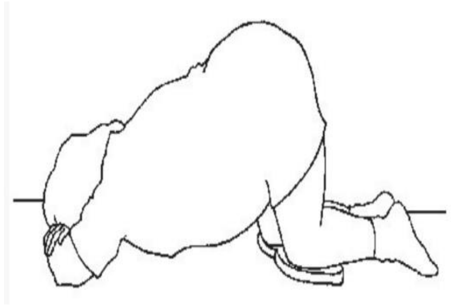

a) Is this positioning favorable for 2nd stage of labour?

- YES
- NO

b) If the patient has her knees positioned apart (hip/belly distance) and her ankles are positioned close to each other and to supination with heels in and toes out. Which part of the pelvis is being opened

- INLET
- OUTLET

c) Is recommended for the patient to maintain this position for minimum 60 minutes?

- YES
- NO

6. Julia is in active labour with regular contracting pattern (3/10), which are easing down. Vaginal findings:

- Dilatation: 6 cm
- Effacement: 100%
- Station: - 1 (II. – III.)

Fetal sagittal suture is not possible to be felt due to caput, but anterior fontanelle is palpable in a posterior position at left lateral side. Which of the following positions could be potentially helpful in case of suspicion malposition, <u>choose all possibilities</u>. **(Asynclitism)**

a) Upright lunge
b) Asymmetrical upright standing
c) Modified upright sitting position/Semisitting
d) Walcher´s.
e) Supine with leg supports.

7. Maria is in the latent phase of labor with the following characteristics:

- Dilation: 4 cm.
- Contractions: Ineffective, every 2-3 minutes.
- Fetal descent: above the 1st Hodge plane.
- Membranes: Intact.
- Pain: Diffuse, mainly in the pubic bone area.
- Monitoring: Intermittent, with FHR of 130 bpm and good variability.

Maria is cooperative, but labor progression is slow. What is the best suggestion to promote fetal engagement? **(Lack of Engangement)**

a) Encourage Maria to remain in a lateral position.

b) Instruct Maria to adopt the Walcher's position ("Opening the Brim") or Flying Cowgirl for at least 3 contractions.

c) Suggest Maria stay in a lateral position with limited mobility, waiting for spontaneous progression.

d) Recommend that Maria remain in a semi-sitting position for comfort, without the need for active movement.

e) Advise immediate analgesia to reduce discomfort and avoid frequent position changes.

8. Ana is in advanced labor, with 9 cm dilation, ruptured membranes, and contractions every three minutes. She is currently in a left lateral position but reports increasing back pain and difficulty coping with contractions. The fetus is in a left occiput posterior (LOP) position. What is the best maternal position to promote physiological birth? **(Occiput Posterior Scenario)**

a) Keep Ana in the left lateral position

b) Encourage hands-and-knees or forward-leaning positions

c) Encourage the right lateral position

d) Instruct Ana to stand or walk to encourage fetal descent

e) Instruct Ana to assume the supine position

9. Cristina has reached complete dilatation (10 cm) and reports an increasing urge to push. She is in a semi-sitting position with contractions occurring every 2 minutes, and fetal descent is at 4th Hodge plane. The fetal heart rate is reassuring. As a midwife, what is the best positioning strategy to support physiological birth? **(No scenario – NSVD)**

a) Encourage Cristina to remain in a semi-sitting position with spontaneous pushing efforts.

b) Instruct Cristina to assume the lithotomy position to allow better visualization of the perineum.

c) Encourage Cristina to adopt an upright squatting position with support to optimize the pelvic outlet.

d) Recommend that Cristina lie in the dorsal position and perform directed pushing with every contraction.

e) Advise Cristina to hold her breath and push forcefully with each contraction to shorten the second stage.

10. Laura is in the active phase of labor with regular contracting pattern (4/10). She is standing with straightened legs, leaning on and is bent over of hospital bed. Vaginal examination findings: Cervical dilation 7-8 cm, 100 % effacement, fetal station -2. No progress in fetal descent has been observed over the past two hours. What is the most appropriate management to facilitate labor progression?

(Arrest of Descent Scenario)

a) Repositioning to an asymmetrical posture, such as a lunge or lateral position with one leg elevated.

b) Maintaining the current position and waiting for spontaneous fetal descent for another 2 hours and reassess the vaginal findings.

c) Initiating active directed pushing to expedite delivery.

d) Immediate administration of oxytocin infusion therapy to enhance uterine contractions.

e) Placing the patient in the supine position with leg supports.
